# Function Forms from the Symmetry Between Order and Disorder

**DOI:** 10.1093/function/zqaa037

**Published:** 2020-12-15

**Authors:** Denis Noble

**Affiliations:** Department of Physiology, Anatomy & Genetics, University of Oxford, OX13PT, UK

As we enter the third decade of the 21st century, we can expect the century to be a great one for the study of function, which is what physiology is about.

First, we will see advances in the age-old symmetry between order and disorder. Order is of course characteristic of physiological control systems. All physiological levels from subcellular, through cellular, tissues, organs, and up to whole-body systems, are forms of organization and are characterized by the constraints they exert on levels below them. Disorder, by contrast, is characterized by stochasticity, first studied in the 19th century as Brownian motion, and eventually explained by Albert Einstein as the result of thermal jiggling together of water and other molecules and particles dissolved or suspended within it. The 20th-century biology saw stochasticity as noise, a nuisance in any communication system. At best, it was a source of novelty in the form of the errors of copying and other random changes that we call mutations. The great quantum mechanics pioneer, Erwin Schrödinger even argued in his book *What is Life?* that living organisms derive “order from molecular level order,” in contrast to physics which, through thermodynamics, is the study of “order from disorder.” In the hands of Watson and Crick, his idea eventually led to the Central Dogma of molecular biology.

Schrödinger was wrong. With rare exceptions (outlier genetic diseases), biology must also create order from disorder. The full reasons are given elsewhere.^1^ I, therefore, predict that the 21st century will witness the demise of the Central Dogma^2^ in its frequent interpretation as excluding the functional reorganization of genomes. It will remain as the more limited statement of the unidirectional templating between DNA sequences and protein sequences, which is precisely what the triplet code is about. It says nothing about the control of mutation rates and genome reorganization through the processes of natural genetic engineering.^3^

Because of this demise, we will see physiology becoming even more relevant to the study of diseases like cancer. A recent symposium on evolution and cancer (https://cancerevolution.org/) featured the revolutionary idea that cancer is best studied as the stochastic development of a new species within the tissues of the organism, which readily explains the rapid radiation of genomic forms in late-stage metastatic cancer, and why aggressive treatment often provokes further rapid mutation in response to the stress on the cancer “species.” If this insight is followed-up there will be a shift in resources toward understanding the role of physiological networks in giving organisms directionality in response to stress. From being a merely passive reaction to stochasticity (which characterizes evolution according to the Modern Synthesis), stochasticity becomes the clay from which creative novelty arises as organisms (or tumors) feel their way forward. The “watchmaker” was not so much “blind” as “one-eyed.”^4^ This insight has important clinical consequences since the treatment of late-stage cancers is not producing great extensions of life. We need to understand the physiological processes that could provide early-stage markers of cancer.

Which brings me to the third field in which physiology will flourish, that of evolutionary biology itself. The replicator–vehicle view of organisms effectively consigned physiology to the study of the mortal vehicle with no role in the genomic evolution of a species. But once we realize that organisms harness stochasticity, physiology returns to playing a role in the evolution of species and their genomes. We will, therefore, see a resurrection of Conrad Waddington’s masterpiece, *The Strategy of the Genes*,^5^ which sees the “landscape of development” lying above the genes and canalizing them toward further functional evolution. A modernized version of his famous landscape diagram is illustrated in the figure.
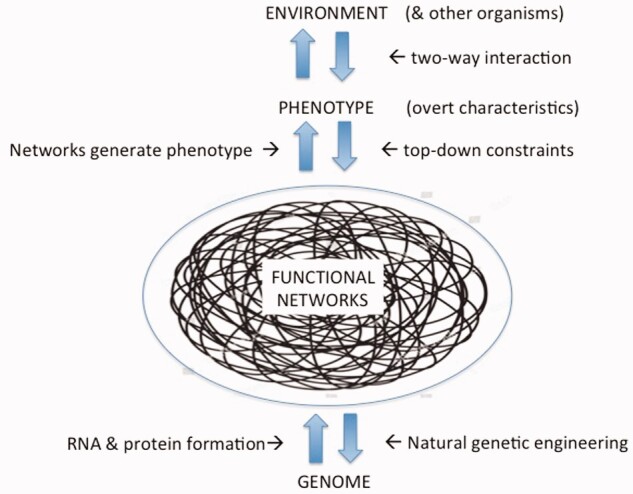

Modern version of Waddington’s 1957 idea of canalisation (harnessing) of genetic variation and effects using natural genetic engineering. All causal arrows are two way since organisms are nested open systems all the way from top to bottom. There is no direct causal link between genotype and phenotype. All effects are mediated by active functional networks. The genome is a passive store of templates for RNA & Protein production. Network robustness explains the very low association scores found in Genome Wide Association Studies. (tangled network from https://www.pinterest.co.uk/pin/821836631980512728/) https://www.shutterstock.com/image-vector/chaos-tangle-circle-doodle-line-chaotic-1055187698

I come to my final prediction. The harnessing of stochasticity is how the immune system achieves its remarkably rapid success in generating new DNA sequences within the coding for the variable part of the immunoglobulins. Hypermutation can increase the natural mutation rate by hundreds of 1000-fold, and precisely in the variable part of the immunoglobulin which determines what shape of antigen it can attack. The immunologist who pioneered this Nobel Prize winning work, Gerald Edelman, then went on to a controversial extension of his idea to the nervous system in the form of his neural selection theory.^6^ In that theory, neuronal stochasticity becomes the clay from which organisms can generate unlimited forms of associative learning.^7^ Conscious intentional behavior may well depend on the neuronal selection ideas that Edelman proposed. His theory was severely criticized by Francis Crick^8^ on the grounds that it does not have the equivalent of a replicator. Edelman called his idea Neural Darwinism, which may have been unfortunate. In a view of biology in which the replicator–vehicle distinction is invalid Crick’s criticism is no longer important. It does not need to involve a replicator. All it requires is a repertoire of behavioral options from which matches to rational behavior can be chosen by the organism.^9^ It is not itself an evolutionary theory.

These then are my “crystal ball” prophecies. If they materialize over the next decades we will witness a great resurrection of the study of function in biology.
